# Frequency of NAT2 and GSTP1 polymorphisms in the Kazakh population

**DOI:** 10.5195/cajgh.2013.83

**Published:** 2014-01-24

**Authors:** Aisha Iskakova, Aliya Romanova, Elena Zholdybayeva, Erlan Ramanculov, Kuvat Momynaliev

**Affiliations:** 1 National Center for Biotechnology, Astana, Kazakhstan; 2 Nazarbayev University, Astana, Kazakhstan

**Keywords:** NAT genes, GSTP genes, genetic polymorphism, Kazakh population

## Abstract

**Introduction:**

Phase II xenobiotic biotransformation enzymes perform detoxification of hydrophilic and often toxic Phase I products through glutathionetransferase (GST), UDP-glucuronosyltransferase (UDF), N-acetyltransferase (NAT) families and other enzymes. GST protein families metabolize a large number of electrophilic xenobiotics, by conjugating fusing them with glutathione. Arylamine-N-acetyltransferase (NAT) catalyzes the acetylation of the aromatic and heterocyclic amines.

**Materials and methods:**

This study assesses the frequency of NAT2 and GSTP1 gene polymorphisms in 326 healthy individuals from different regions of Kazakhstan by using Real-Time PCR and direct sequencing methods.

**Results:**

The allele frequencies were calculated for NAT2*5 (0.54) and GSTP1 (0.27). GSTP1 alleles were in the Hardy–Weinberg equilibrium (p > 0.05), while NAT2*5 (p = 0.00) were not. The population differences between North, Northeast and South Kazakhstan regions were also analyzed. No statistically significant differences in the frequency of genotypes were found.

**Conclusion:**

Allelic polymorphisms of NAT2*5 and GSTP1 genes greatly varied indifferent populations. The Kazakh population was significantly different from the Asian, Caucasoid, African-American and Hispanic populations by NAT2*5 and GSTP1 genes. Allelic variants of the NAT2*5 had a low frequency in Asian populations. Allelic frequency in other world populations varied from 30 to 50%. The differences between Kazakh (0.54) and the world population were statistically significant (p < 0.05). The frequency of GSTP1 (rs1695) in the African American population was 42%. The frequency of GSTP1 in Asian populations varied from 11% to 23%. The frequency in Caucasoid populations was around 30%. The differences between Kazakh population (0.27) and other populations selected were statistically significant (p < 0.05).

The study of mutations in GSTP1 and NAT2 genes is necessary in assessing the risk of the development of various diseases, such as cancer. Information on allelic polymorphisms might also be useful for personal perscriptions such as cyclophosphamide, cisplatin, methotrexate, isoniazid, pyrazinamide, and rifampin.

